# Levels of Aqueous Humor Trace Elements in Patients with Non-Exsudative Age-related Macular Degeneration: A Case-control Study

**DOI:** 10.1371/journal.pone.0056734

**Published:** 2013-02-15

**Authors:** Anselm G. M. Jünemann, Piotr Stopa, Bernhard Michalke, Anwar Chaudhri, Udo Reulbach, Cord Huchzermeyer, Ursula Schlötzer-Schrehardt, Friedrich E. Kruse, Eberhart Zrenner, Robert Rejdak

**Affiliations:** 1 Department of Ophthalmology, University Hospital of Erlangen, Erlangen, Germany; 2 Department of General Ophthalmology, Medical University of Lublin, Lublin, Poland; 3 Institute of Ecological Chemistry, Helmholtz Zentrum München, Neuherberg, Germany; 4 Bio-Inorganic Chemistry, University of Erlangen-Nürnberg, Erlangen, Germany; 5 Department of Public Health and Primary Care, Trinity College Dublin, Dublin, Ireland; 6 Ophthalmic Research, Centre for Ophthalmology, Tübingen, Germany; 7 Department of Experimental Pharmacology, Medical Research Centre, Polish Academy of Sciences, Warsaw, Poland; Case Western Reserve University, United States of America

## Abstract

Trace elements might play a role in the complex multifactorial pathogenesis of age-related macular degeneration (AMD). The aim of this study was to measure alterations of trace elements levels in aqueous humor of patients with non-exsudative (dry) AMD.

For this pilot study, aqueous humor samples were collected from patients undergoing cataract surgery. 12 patients with dry AMD (age 77.9±6.62, female 8, male 4) and 11 patients without AMD (age 66.6±16.7, female 7, male 4) were included. Aqueous levels of cadmium, cobalt, copper, iron, manganese, selenium, and zinc were measured by use of Flow-Injection-Inductively-Coupled-Plasma-Mass-Spectrometry (FI-ICP-MS), quality controlled with certified standards.

Patients with AMD had significantly higher aqueous humor levels of cadmium (median: 0.70 µmol/L, IQR: 0.40–0.84 vs. 0.06 µmol/L; IQR: 0.01–.018; p = 0.002), cobalt (median: 3.1 µmol/L, IQR: 2.62–3.15 vs. 1.17 µmol/L; IQR: 0.95–1.27; p<0.001), iron (median: 311 µmol/L, IQR: 289–329 vs. 129 µmol/L; IQR: 111–145; p<0.001) and zinc (median: 23.1 µmol/L, IQR: 12.9–32.6 vs. 5.1 µmol/L; IQR: 4.4–9.4; p = 0.020) when compared with patients without AMD. Copper levels were significantly reduced in patients with AMD (median: 16.2 µmol/L, IQR: 11.4–31.3 vs. 49.9 µmol/L; IQR: 32.0–.142.0; p = 0.022) when compared to those without. No significant differences were observed in aqueous humor levels of manganese and selenium between patients with and without AMD. After an adjustment for multiple testing, cadmium, cobalt, copper and iron remained a significant factor in GLM models (adjusted for age and gender of the patients) for AMD.

Alterations of trace element levels support the hypothesis that cadmium, cobalt, iron, and copper are involved in the pathogenesis of AMD.

## Introduction

Age-related macular degeneration (AMD) is a leading cause of irreversible vision loss in industrialized countries. The exact etiology of this complex multifactorial disease is unknown, but is believed to involve interaction of genetic and environmental factors [1].

There is some evidence that trace elements might play a role in the pathogenesis of AMD. Iron is a potent generator of reactive oxygen species (ROS), whose generation within mitochondria and lysosomes may promote cell death [2]. Iron has been suggested as a source of oxidants in AMD, as AMD-affected maculas were found to have higher concentrations of iron than healthy age-matched maculas [3]. Iron was found in the retinal pigment epithelium (RPE) and Bruch's membrane in early AMD, geographic atrophy, and exudative AMD.

Tobacco smoking is one of the few established environmental risk factors for AMD [4]. Recent research has implicated cadmium as a possible contributor to smoking-related AMD. It was reported that cadmium levels in retinal tissues were approximately twice as high in smokers as in nonsmokers [5]. In addition, higher urinary cadmium levels, indicating a higher total body burden of cadmium, were found in smokers who had AMD compared to smokers who did not have AMD [6]. These findings raised the possibility that cadmium exposure might play a role in tobacco-related AMD. Cadmium is a potent inflammatory agent and increases oxidative stress [7]. Oxidative stress and inflammation have been linked to AMD [8].

Copper and zinc play vital roles in retinal function and are essential for antioxidant defense mechanisms [9], which are important for the survival of the retina, who is routinely exposed to high levels of oxidative stress from light and metabolic processes. Both copper and zinc are known to be necessary for the visual cycle and photoreceptor survival. These metals act as co-factors for the antioxidant enzyme copper-zinc superoxide dismutase, which catalyzes the conversion of superoxide to oxygen and hydrogen peroxide (H_2_O_2_). Copper and zinc also stimulate protective cellular stress-signaling pathways and stabilize proteins, making them less vulnerable to oxidation.

Cobalt is an essential trace element for humans, but becomes toxic at high concentrations [10]. Selenium-containing glutathione peroxidase is an important part of the cellular antioxidative system, and selenium itself is widely used in dietary supplements. Polymorphisms of manganese superoxide dismutase (MnSOD) genes may be associated with the development of AMD [11].

Based on this evidence, the aim of this study was to measure alterations of aqueous humor trace elements levels in patients with AMD.

## Patients and Methods

### Ethics Statement

The present prospective case-control study was approved by the Institutional Review Board of the University of Erlangen-Nürnberg. After detailed explanation of the purpose and methods of the study, written and informed consent was obtained from the subjects, who were all German, before participating. The study was conducted in adherence to the tenets of the Declaration of Helsinki.

### Patients and design

At the University Eye Hospital Erlangen, patients who underwent cataract surgery as inpatients were collected using a convenient sampling approach. A priory power analysis showed that a sample size of 10 to 15 per group would be sufficient for detecting differences in trace element levels in the aqueous humor (power 80%, significance level 5%). Of a total of 90 patients undergoing surgery in december of 2010, 12 patients with dry AMD fulfilled the inclusion criteria (age 77.9 years; SD: 6.6; 8 females, 4 males), and 11 patients without AMD (age 66.6 years; SD: 16.7, female 7, male 4) were matched to these patients. All participants were of Caucasian race.

All controls and patients were thoroughly examined by slit lamp inspection, applanation tonometry, fundoscopy, and gonioscopy. Criteria for AMD diagnosis were the presence of drusen and/or irregularities of retinal pigment epithelial cells. However, patients with signs of exsudative AMD were excluded. Controls (cataracts) had no signs of AMD. In order to match the groups as closely as possible, a detailed medical history was obtained and controls were matched to cases by demographic, clinical, nutritional and lifestyle data known to affect trace element levels. Nutritional and lifestyle status were briefly assessed using the SGNA-test (subjective global nutritional assessment-test). Patients with hypovitaminoses were excluded (data not shown), so that all participants (AMD patients and controls) were classified as well-nourished. Exclusion criteria were: medical history of major systemic illness (vasculitis, renal and hepatic disease), gastrointestinal malabsorption, psychiatric illness, hypothyroidism, severe psoriasis, malignant neoplasias, evidence of chronic alcohol abuse during the past half year, cigarette smoking, as well prior ocular surgery, a history of ocular inflammation, diabetic retinopathy, myopia, retinal occlusive disease, and rubeosis iridis. We excluded patients taking vitamin supplements or other medications which affect trace element concentrations, such as fibrates, carbamazepine, phenytoin and antifolates (methotrexate and trimethoprim). Demographical data, medical history and systemic medication are summarized in tables 1 and 2. Age and gender as potentially confounding factors were accounted for by inclusion in the general linear model.

**Table 1:** Demographical data and medical history of patients in the AMD and the control group.

	AMD group	Control group
No. of patients	12	11
Age (years)	77.9±6.62	66.6±16.7
Male∶female	8∶4	7∶4
**General Medical History**		
Arterial hypertension (no. of patients)	8	7
Coronary artery disease	2	2
Cardiac arrythmia	1	2
Hyperlipidemia	3	3
Hyperuricemia	1	2
Diabetes mellitus	2	1
Struma	1	3
Osteoporosis	2	1
Others (asthma, hirsutism, arthrosis, depression, menopause, COPD, peripheral arterial disease)	5	4

**Table 2:** Systemic medication of patients in the AMD and the control group.

	AMD group	Control group
**Systemic Medication**		
Number drugs (mean ± SD)	3.25±3.3	3.0±2.6
Antihypertensive drugs (no. of patients)	8	7
Antiarrhythmic drugs	0	2
Diuretic/allopurinol	3	3
Nitrates	2	1
Hormones (estrogen, anti-androgen, thyroxin, steroid)	2	6
Antidepressant	3	1
Statin	3	3
Bisphosphonate	1	0
Proton pump inhibitor	1	1
Coumarine derivative	1	0
Antiplatelet agent	2	1
Antidiabetic drug	2	1
Methylxanthine	0	2
Others (NSAID, tramadolol, herbal)	2	3

### Laboratory analysis

All patients with AMD and controls underwent cataract surgery with phacoemulsification. Aqueous humor samples were obtained intraoperatively before fashioning of the corneal tunnel. 100 to 150 µl of aqueous humor was withdrawn through an ab-externo limbal paracentesis site using a 27-gauge needle on a tuberculin syringe, with special care to avoid blood contamination. The samples were immediately frozen in liquid nitrogen and stored in a deep freezer at −80°C until biochemical analysis. Cadmium, cobalt, copper, iron, manganese, selenium, and zinc were measured using Flow-Injection-Inductively-Coupled-Plasma-Mass-Spectrometry (FI-ICP-MS) following certified standards.

### Elemental analysis

Due to the small sample volumes available, a combination of flow injection sample introduction with ICP-MS detection was used. The flow injection sample introduction was conducted by coupling a Knauer 1100 Smartline inert Series HPLC system to the ICP-MS. The HPLC system was equipped with an electronic valve with a 25 µl injection loop (Perkin Elmer, Rodgau-Jügesheim, Germany). The flow rate was 1 ml/min of Milli-Q water. The outlet of the injector port was directly connected to the nebulizer of the ICP-MS by PEEK (polyetheretherketone) capillary tubing (0.125 cm i.d., ca. 80 cm). Instrumental settings for ICP-MS are shown in table 3. Calibrations were performed with daily fresh prepared standard solutions in the ranges 0–1000 ng/L for Cd, Co, Hg, Mn, Pb, Se or 0–100 µg/L for Cu, Fe, Zn. R^2^ values were 0.989 for Zn and better than 0.999 for the other elements. For each element a 5-point calibration curve was achieved and sample peak areas were related to respective element calibrations.
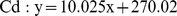


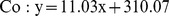


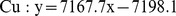


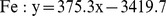


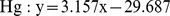


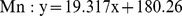


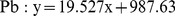


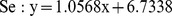


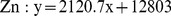
Triplicate measurements were performed for each sample. For quality control, standard solutions and blanks were measured periodically between the samples. Data processing of FI-ICP-MS signals was accomplished by exporting the element data files from the ICP-MS control software and integrating FI-element peaks with the Knauer HPLC software “Clarity”.

**Table 3:** Instrumental settings for Inductively-Coupled-Plasma-Mass-Spectrometry (ICP-MS).

Instrument	Perkin Elmer Sciex ELAN DRC II, Toronto, Canada
**Plasma conditions**	
Rf power (W)	1250
Plasma gas flow (L/min)	15
Auxiliary gas flow (L/min)	1.1
Nebulizer gas flow (L/min)	0.83, daily optimized
**Mass spectrometer settings**	
Dwell time (ms)	50
Sweeps per reading	3
Readings per replicate	1000
Autolens	On
Isotopes monitored	^56^Fe,^114^Cd,^59^Co,^66^Zn,^63^Cu,^55^Mn,^80^Se
Rejection parameter q	0.25

### Statistical analysis

The distribution of trace elements in aqueous humor was considered skewed. Heteroskedasticity was also observed. Statistical differences between levels of trace elements in aqueous humor in patients with and without AMD were assessed using the Mann-Whitney U test. For each trace element, a General Linear Model was calculated. AMD and gender were entered as factors in each model, with age as a covariate. Concentrations of all trace elements were transformed to normality using a natural logarithm transformation. All statistical tests were two-tailed. P-values were adjusted using a Bonferroni correction. Statistical analyses were performed using InStat (GraphPad Software, Inc. CA, USA) and PASW Statistics 18 (IBM, Somers, NY, USA).

## Results

**Figure 1:**
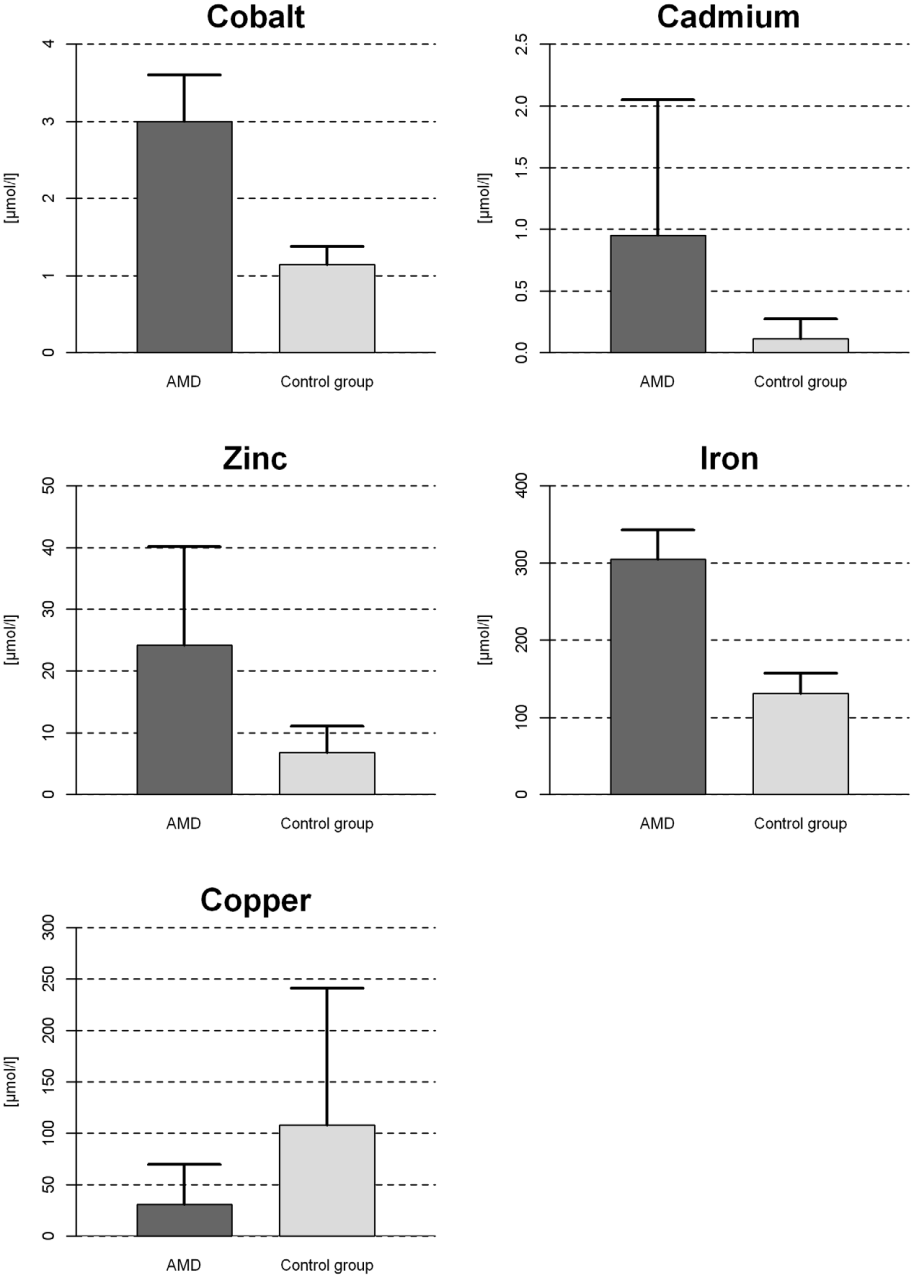
Differences in the levels of aqueous humor trace elements (µmol/L) in group of patients with AMD and control group.

Patients with AMD had significantly higher levels of cadmium (median: 0.70 µmol/L, IQR: 0.40–0.84 vs. 0.06 µmol/L; IQR: 0.01–.018; p = 0.002), cobalt (median: 3.1 µmol/L, IQR: 2.62–3.15 vs. 1.17 µmol/L; IQR: 0.95–1.27; p<0.001), iron (median: 311 µmol/L, IQR: 289–329 vs. 129 µmol/L; IQR: 111–145; p<0.001), and zinc (median: 23.1 µmol/L, IQR: 12.9–32.6 vs. 5.1 µmol/L; IQR: 4.4–9.4; p = 0.020) when compared with patients without AMD. Copper level was significantly reduced in patients with AMD (median: 16.2 µmol/L, IQR: 11.4–31.3 vs. 49.9 µmol/L; IQR: 32.0–.142.0; p = 0.022) when compared to those without (see Fig. 1). No significant differences in the aqueous humor levels of manganese and selenium were observed between patients with or without AMD.

A General Linear Model (GLM) with age as covariate and gender and AMD as factors was calculated for each trace element (transformed to normality using a logarithm transformation with the natural logarithm).After an adjustment for multiple testing, AMD was a significant factor in GLM models regarding cadmium, cobalt, copper, and iron. Details of mean values and standard deviation of aqueous humor in patients with and without AMD, including test statistics for the GLM models can be found in table 4 (Mean ± standard deviation are displayed; the displayed p-values are Bonferroni-corrected).

**Table 4:** Trace elements in patients with age-related macular degeneration (AMD) and patients with cataract and without AMD (control group).

	AMD	Patients without AMD	GLM test value	Significance
Cadmium (µmol/L)	0.95±1.09	0.12±0.16	F = 26.15	p<0.001
Cobalt (µmol/L)	3.00±0.61	1.14±0.24	F = 48.88	p<0.001
Copper (µmol/L)	30.7±39.2	107.9±133.5	F = 23.24	p<0001
Iron (µmol/L)	305.1±37.5	131.3±26.0	F = 67.2	p<0.001
Manganese (µmol/L)	2.20±1.01	2.24±1.69	F = 1.10	not significant
Selenium (µmol/L)	5,92±1,90	7,66±4,12	F = 6.40	not significant
Zinc (µmol/L)	24.17±15.92	6.78±4.32	F = 0.50	not significant

Mean ± standard deviation are displayed; p-value (2-tailed) is Bonferroni-corrected. The Mann-Whitney U test was used.

## Discussion

In the present study we observed significantly higher levels of cadmium, cobalt, iron, and zinc, while copper levels were reduced in the aqueous humor of patients diagnosed with AMD when compared with patients without AMD. Manganese and selenium levels showed no significant differences between the two groups. After adjustment for multiple testing; cadmium, cobalt, copper and iron remained a significant factor in age- and sex adjusted GLM models for AMD. We are unaware of any previous studies describing trace element concentrations in the aqueous humor of AMD patients, and could not find any respective reference in a computerized search utilizing Medline.

There is evidence that oxidative stress is involved in the formation of drusen and in the pathogenesis and progression of AMD. Hydroxyl radicals are extremely reactive, causing lipid peroxidation, DNA strand breaks, and degradation of biomolecules. Particularly in photoreceptors, where there is a high oxygen tension and high concentration of easily oxidized polyunsaturated fatty acids, reactive oxygen species must be tightly controlled to avoid oxidative damage. Oxidative stress and inflammation have both been linked to AMD [8]. In the Fenton reaction, iron reacts with hydrogen peroxide (H_2_O_2_) to produce hydroxyl radicals, the most reactive and toxic of the reactive oxygen species (ROS). Retinal degeneration has also been observed in hereditary disorders resulting in iron overload, including aceruloplasminemia, hereditary hemochromatosis, pantothenate kinase associated neurodegeneration, and Friedreich's Ataxia. AMD-affected maculas contained more iron than healthy age-matched maculas 3]. Our results of increased iron in the aqueous humor of AMD patients seem to confirm a role of this metal in the pathogenesis of AMD.

Another trace metal known to induce oxidative stress with higher concentration in aqueous humor of AMD patients is cadmium. The biologically significant ionic form of cadmium, Cd^2+^, binds to many bio-molecules and these interactions underlie the toxicity mechanisms of cadmium. Some of the molecules specialized in the handling of alkaline earth (Mg^2+^, Ca^2+^) and transition metal ions (e.g. Zn^2+^, Cu^2+^, Fe^3+^/^2+^) should be particularly sensitive to the presence of Cd^2+^, because they enclose cationic sites to which the toxic metal can bind [12]. Metallothionein is an important intracellular storage protein for zinc and copper, and its synthesis is decreased in oxidative stress [13]. Considering the tight binding of Cd^2+^ by metallothionein and the sensitivity of the expression of its genes to stressful conditions, this protein may mediate cadmium toxicity in various ways. These include decrease of the zinc buffering ability of cells in different compartments, changing of the dynamics of zinc exchanges, and decrease of the cellular antioxidant defense [14]. Exposure to cadmium perturbs the homeostasis of other metals, and, reciprocally, this effect depends on the body status of other essential metals such as iron and zinc. This interaction is regularly observed in a variety of conditions [15]. Zinc often affords protection against cadmium toxicity, and cells adapted to high zinc concentrations display changed cellular handling homeostasis of cadmium, manganese, and calcium [16].

Zinc and copper are cofactors of metalloenzymes that play a critical role in cell structure and function. Among these enzymes is copper-zinc superoxide dismutase, which regulates oxidative stress in the RPE. Studies show that zinc plus copper supplementation decreases the risk of progression of AMD [17]. Aqueous zinc levels were increased while copper concentrations were reduced in our study. A decrease of copper and zinc concentrations in the RPE and choroid complex of AMD-affected subjects has also been reported [18]. It was shown that average levels of zinc and copper in the neural retina were lower in aged eyes than in young eyes, whereas increase in these metal levels occurred in the choroid; also correlation between cadmium accumulation and increase in zinc and copper levels in males was observed [5]. High zinc concentration was also shown in macular sub-RPE deposits of patients with AMD [19]. Zinc may also be released from intracellular deposits of the RPE and photoreceptors due to apoptosis of these cells. These mechanisms may lead to elevated extracellular levels of this metal despite its suspected intracellular deficiency. As AMD was not a significant factor in a general linear model regarding zinc, the possible role of this trace element in the pathogenesis of AMD remains uncertain from the results of this study.

We have reported elevated cobalt levels in patients with AMD. Cobalt can cause DNA fragmentation and activation of caspases, increased production of reactive oxygen species, and beta amyloid secretion [20]. A significant depletion of intracellular Zn^2+^ and Mg^2+^ after CoCl_2_ exposure has been described [21]. A substitution of magnesium ions by cobalt ions may result in the interruption of ATPases and the energy balance of the cell [22]. Ionic cobalt (Co^2+^) is known to exert hypoxia-like responses by stabilizing the alpha subunit of the hypoxia inducible transcription factor (HIF1) [23]. This results in changed gen transcription of encoding proteins that play key roles in angiogenesis, glucose and energy metabolism, cell survival and proliferation, iron metabolism, and vascular functions [24]. Comparative gene expression studies showed HIF1-mediated responses to be similar for hypoxia and CoCl_2_ exposure [25]. Although all these mechanisms have been described for the immediate toxicity of much higher cobalt concentrations than reported in our study, they might also play a role in a long-standing exposure to lower concentrations. We could not find any published data on an involvement of cobalt toxicity in AMD pathogenesis.

The major limitation of this pilot study is the relatively small sample size. Generally, a bias may be introduced by a convenient sampling approach; however, this seems unlikely in the present study due to the high prevalence of cataract in the general population and the case-control design.

Our findings of significant alterations in aqueous humor metal levels in AMD-affected eyes support the hypothesis that their dysregulation may be involved in the pathogenesis of AMD. Knowledge of trace elements distribution, metabolism and toxicity will help to understand their role in the pathogenesis of AMD. Properly designed studies implementing biologically relevant intracellular and extracellular trace elements concentrations in different ocular tissues are required. Improved knowledge of the essential metals homeostasis and combination of this data with other aspects of AMD may help to tackle treatment of this disease.
